# A comparison of AAV-vector production methods for gene therapy and preclinical assessment

**DOI:** 10.1038/s41598-020-78521-w

**Published:** 2020-12-09

**Authors:** Marcus Davidsson, Matilde Negrini, Swantje Hauser, Alexander Svanbergsson, Marcus Lockowandt, Giuseppe Tomasello, Fredric P. Manfredsson, Andreas Heuer

**Affiliations:** 1 Department of Neurobiology, Barrow Neurological Institute, Phoenix, AZ USA; 2 Behavioural Neuroscience Laboratory, Department of Experimental Medical Sciences, Lund University, Lund, Sweden; 3 Neural Plasticity and Repair, Department of Experimental Medical Sciences, Lund University, Lund, Sweden; 4 CNS Gene Therapy, Department of Experimental Medical Sciences, Lund University, Lund, Sweden

**Keywords:** Genetic vectors, Molecular neuroscience

## Abstract

Adeno Associated Virus (AAV)-mediated gene expression in the brain is widely applied in the preclinical setting to investigate the therapeutic potential of specific molecular targets, characterize various cellular functions, and model central nervous system (CNS) diseases. In therapeutic applications in the clinical setting, gene therapy offers several advantages over traditional pharmacological based therapies, including the ability to directly manipulate disease mechanisms, selectively target disease-afflicted regions, and achieve long-term therapeutic protein expression in the absence of repeated administration of pharmacological agents. Next to the gold-standard iodixanol-based AAV vector production, we recently published a protocol for AAV production based on chloroform-precipitation, which allows for fast in-house production of small quantities of AAV vector without the need for specialized equipment. To validate our recent protocol, we present here a direct side-by-side comparison between vectors produced with either method in a series of in vitro and in vivo assays with a focus on transgene expression, cell loss, and neuroinflammatory responses in the brain. We do not find differences in transduction efficiency nor in any other parameter in our in vivo and in vitro panel of assessment. These results suggest that our novel protocol enables most standardly equipped laboratories to produce small batches of high quality and high titer AAV vectors for their experimental needs.

## Introduction

Viral vectors are viruses that have been altered to be used as vehicles for gene delivery^1–3^. These complex macromolecules have evolved over eons to enter cells and to utilize the cells machinery to replicate. This trait has been reappropriated by scientists for various applications. For safety reasons naturally occurring viruses have been rendered replication deficient, so while they retain their ability to infect cells and express transgenes, they cannot re-assemble into infectious particles. Viral vectors as means of gene-delivery vehicles are implemented in virtually all areas of medical research where they are used to induce or silence gene expression^3–6^. For the former, a transgene of interest is expressed in a cell to study its function, to label the cell for anatomical studies^7–10^, or to overexpress artificial receptors to perturbate the cells activity pattern (e.g. optogenetics/chemogenetics)^11–15^. For the latter, genes can be selectively knocked out using CRISPR/Cas technology or silenced using RNAi strategies^16–24^. The adeno-associated viruses are one of the most common tools for transgene delivery. The AAVs are part of the parvovirus family and consist of a single stranded DNA virus and have a packaging capacity of about 4.7 kb^6,25–27^. Their main advantage is their low immunogenicity and the property that they remain episomal, therefore causing a low risk of mutagenesis. The episomal nature of the recombinant genome does make it sensitive to dilution via cell division, however, active AAV genomes have been found in human post-mortem tissue 10 years following delivery to non-dividing cells^28^. Although the cargo capacity of AAVs is comparatively small compared to other viral based expression systems^29,30^, co-infection rates are relatively high. Cell type specificity can be achieved by serotype selection (tissue propensity), choice of promoter (e.g. neuron specific), or dependent gene switches (dre, flp, cre)^26,31,32^. To achieve faster transgene expression self-complementary (sc) AAVs have been developed through which higher and faster transgene expression can be achieved, albeit for the price of halving the packaging capacity^33^. Many recent developments aim to generate AAV-vectors that have a higher propensity for certain neuronal populations, broader diffusion, or directional transport^9,10,34–38^. AAVs have been used for gene therapy clinical trials for a broad range of disorders^39–44^ and there are currently 215 clinical trials ongoing, or completed using the AAV platform (www.clinicaltrials.gov/), at the time of writing. Promising targets for gene therapy include Leber’s congenital amaurosis (LCA, inherited blindness)^45^, retinal dystrophy^46,47^, haemophilia A and haemophilia B^48^, DMD, SMA^49^, but also neurodegenerative disorders such as Alzheimer’s disease, Parkinson’s disease and Huntington’s disease^50^.

Although AAV-vectors are used commonly in the scientific community, most often they are obtained from commercial suppliers. Depending on whether a standard vector is necessary, or a more complex, individual tailored vector is warranted, there can be a substantial cost and/or time delay for production. This can be prohibitive, as in an explorative phase of research, it is quite common to test many constructs in parallel, before committing to one for a large-scale production. We recently published a protocol which allows for the simple and efficient production of high-titer AAV using standard equipment that is available in most molecular laboratories^51^ based on the original use of Chloroform to purify AAV vectors^52^. We have used this production method successfully to overexpress transgenes for cell identification and activity perturbation^7,10–12^ in the rodent brain. In the current manuscript we compare the gold-standard of iodixanol viral vector production^53,54^ used by most commercial suppliers (iodixanol-based) with our novel protocol (chloroform-based), which allows for a simpler and faster production of high titer AAV vectors. We report here that vectors from both production methods display similar efficiencies in transducing cells in vitro as well as in vivo. Neither production method, at a titer of 1 × 10^12^ genome copies/mL (gc/mL), did result in any obvious inflammatory response nor loss of TH immunoreactive (TH-ir) neurons in the substantia nigra, at the timepoint assessed. These results combined suggest that chloroform-based AAV vector production for preclinical analysis is equipotent to Iodixanol-based production methods.

## Methods

### Experimental outline

**Figure 1:**
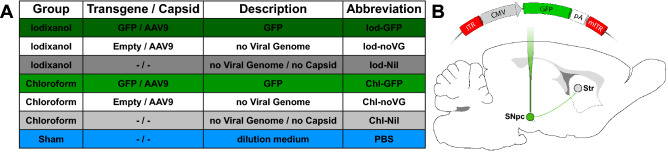
Experimental outline: (**A**) Description of the seven experimental groups. (**B**) Schematic of the self-complimentary (sc) viral genome expressing GFP under the CMV promoter injected into the midbrain of rats. *GFP* green fluorescent protein, *Iod* iodixanol, *Chl* chloroform, *SNpc* Substantia nigra pars compacta, *Str* striatum, *ITR* inverted terminal repeat, *CMV* cytomegalovirus promoter, *pA* polyadenylation site.

The experimentation utilized 53 female Sprague–Dawley rats (Janvier) divided into seven experimental groups (Fig. 1A). For injections we compared the products according to Fig. 1A of iodixanol (Iod) to choloform (Chl) based production protocols. All rats received unilateral injections of either a AAV9 expressing GFP (Iod/Chl-GFP), a AAV-vector without a viral genome (Iod/Chl-noVG), the supernatant from production line cell culture (not containing viral capsid plasmid; Iod/Chl-Nil), or PBS, into the right substantia nigra pars compacta (SNpc; Fig. 1B). The groups were chosen to compare the actual transgene expression (Iod-GFP vs Chl-GFP), to control for the differences in production methods (GFP vs noVG/Nil, as well as for the effect of surgery (PBS). All rats were subjected to the amphetamine-induced rotation test (see below) 5 weeks post-surgery to assess potential degeneration of the dopaminergic nigrostriatal neurons. Six weeks post-AAV injection all rats were sacrificed, and their brains processed for immunohistochemical analysis.

### AAV vector production

#### Iodixanol gradient ultracentrifugation

AAV was produced as previously described^53–55^. In brief, the sc-CMV-GFP genome was packaged into AAV9 via PEI transfection. HEK293 cells were triple transfected with sc-CMV-GFP, pAAV2/9n and the helper plasmid pXX6. AAVs were purified using an iodixanol ultracentrifugation gradient and concentrated in concentration columns. For the Iod-production, six T500 flasks were used, resulting in 200 µL purified AAV vector with a titer of 2.4 × 10^13^ gc/mL.

#### Chloroform precipitation

AAV was produced as previously described^51,52^. In brief, the sc-CMV-GFP genome was packaged into AAV9 via PEI transfection. HEK293 cells were triple transfected with sc-CMV-GFP, pAAV2/9n and the helper plasmid pXX6. AAVs were harvested 72 h post-transfection using polyethylene glycol 8000 (PEG8000) precipitation and chloroform extraction followed by PBS exchange in concentration columns. For the Chl-production, two T175 flasks were used, resulting in 50 µL purified AAV vector with a titer of 3.0 × 10^13^ gc/mL. For the noVG conditions we omitted the ITR-containing sc-CMV-GFP plasmid and for the Nil condition we omitted the capsid-containing pAAV2/9n plasmid.

### AAV titration

Purified AAVs were titered using droplet digital PCR^56^ with primers specific for the ITRs (forward primer 5′-CGGCCTCAGTGAGCGA-3′ and reverse primer 5′-GGAACCCCTAGTGATGGAGTT-3′). Titers were normalized to 1 × 10^12^ gc/mL using PBS^Mg++/Ca++^.

### Research animals

All experiments conducted in the present study were carried out in accordance with relevant guidelines and regulations (Jordbruksverket). All experimental protocols were approved by the local ethical committee at Lund University (M8343/17). In the present study 53 female rats of the Sprague–Dawley strain were purchased from Janvier and housed in standard laboratory cages in groups of 3–4 rats per cage with access to food and water ad libitum. The animals were randomly assigned to groups (Sham group, n = 5 rats; remaining groups n = 8 rats).

### Surgeries

Intracerebral injections were performed via stereotaxic injections as described elsewhere^57^. In brief, glass microcapillaries were mounted on 10 µL Hamilton syringes and secured on a stereotaxic arm. Anaesthesia was induced with a 5% Isoflurane/O_2_ mixture and maintained at 1.5–2% thereafter. A small burr hole was drilled at the injection site and the injections were targeted to the following coordinates (in mm, from Bregma): AP: − 5.4, ML: − 2.3, DV − 7.5, with the incisor bar set at − 4.5 below the intra-aural line. A total volume of 2 µL was injected at a speed of 0.25 μL/min with an additional 4 min allowed for diffusion before carful retraction of the needle and suturing of the animal. The AAV-vectors were matched to a titer of 1 × 10^12^ genome copies (gc/mL) with PBS, and the noVG/Nil/PBS were injected undiluted.

### Behaviour

To assess behavioural changes due to potential neurodegeneration of midbrain dopaminergic neurons we subjected all animals to the amphetamine-induced rotation test^58^. In brief, all rats were injected with 2.5 mg/kg d-amphetamine i.p. and placed in automated rotometer bowls which were modelled after the design of Ungerstedt^59,60^. Full body turns in either directions were counted for 90 min in 1 min intervals. The data is presented as a net rotation score averaged over the total duration of the test.

### Protein blotting

We investigated the presence of the three capsid proteins VP1, VP2, and VP3. In brief, AAV samples were denatured and run on a 4–12% Bis–Tris gel. Membranes were blotted using the semi-dry method^61^. Blocking was performed for 1 h at RT. The primary antibody (anti-AAV VP1/VP2/VP3, B1, mouse monoclonal antibody (Progen, 61058)) was used at 1:250 dilution with incubation at 4 °C overnight. As secondary anti-Rabbit IgG HRP linked F(ab')2 at a 1:2000 dilution (GE healthcare NA9340-1 ml) was used at RT for 1 h. Amersham ECL prime by GE healthcare was used as detection reagent. For visualization of total protein contents of virus batches, we used Coomassie staining. Briefly, gels were run as for Western blots. They were then stained using 0.1% Coomassie Brilliant Blue R-250, 40% Methanol and 10% Acetic acid at RT overnight. Following staining they were destained using 40% Methanol and 10% Acetic acid until bands were visible. Each well was loaded with 5 × 10^9^ gc for the Iod-GFP and Chl-GFP conditions. Since we could not determine the gc for batches without the transfer-plasmid, we performed equal dilutions within the same production method.

### Perfusion

Six weeks post AAV vector-injection all rats were deeply anaesthetized using 0.2 mL Sodium Pentobarbital until the breathing reflex ceased and the rat was unresponsive to a foot pinch and stimulation of the eye-blink reflex. The brains were then perfused via injections of approximately 150 mL 0.09% saline through the heart followed by 250 mL ice-cold 4% paraformaldehyde (PFA, pH = 7.34) solution. The brains were stored in 4% PFA solution for an additional 24 h for post-fixation before being transferred into a 30% sucrose solution where they were kept for 48 h/until sunk. Brains were thereafter stored at − 20 °C until further processing.

### Immunohistochemistry

Immunohistochemical stainings were performed as described previously^7,57^. In brief, cryopreserved brains were cut on a freezing sledge microtome (Leica) into coronal 1:12 series at a thickness of 40 μm and stored in antifreeze solution at − 20 °C until further processing. Primary-secondary concentrations pairings used in the present study can be found in Table 1. In brief, sections were washed three times in KPBS (NaCl 137 mmol/L, KCL 2.7 mmol/L, Na_2_HPO_4_ 10 mmol/L, KH_2_PO_4_ 1.8 mmol/L, pH = 7.4) and then endogenous peroxidase was quenched for 10 min (10% H_2_O_2_, 10% Methanol, 80% KPBS) before being incubated in 5% serum (matched to the species the secondary antibody was raised in, see Table 1) in TXTBS (TBS + 0.2% Triton X-100, pH = 7.4) for 1 h. Sections were thereafter incubated in primary antibody in 5% serum overnight at room temperature. The primary antibody was removed by washing two times in KPBS and then incubated for 1 h in 5% serum. After this all sections were incubated in secondary antibody solution in 5% serum for an additional hour. The antibody reaction for all non-flourescent secondary antibodies is avidin–biotin based. The secondary antibody was removed by three KPBS washes and sections were incubated in ABC solution (Vectorlabs) for 1 h. After three additional KPBS washes the sections were incubated for 2 min in a 3,3′-Diaminobenzidine-solution before developing the substrate. The sections were mounted on gelatin coated glass slides and de-hydrated through incubation in an increasing series of ethanol, removing lipids by Xylene and finally coverslipped using DPX. For visualizing fluorescent secondary antibodies, we omitted the quenching step before primary antibody incubation, and we visualized the primary antibody by incubation in fluorophore-conjugated secondary antibodies. Fluorescent sections were mounted directly onto gelatin-coated glass slides and coverslipped after 10–15 min using PVA-DABCO. The specific primary/secondary antibodies used in the present study are given in the table below (Table 1).

**Table 1:** Antibodies used in the current study for immunohistochemical analysis.

1° AB	Species	Cat. no.	Conc. 1°	Serum	2° AB	Cat. no.	Conc. 2°
GFP	Chicken	A10262 (Thermofisher)	1:10,000	Goat	anti-chicken	BA9010 (Vector)	1:200
GFP	Chicken	A10262 (Thermofisher)	1:10,000	Donkey	anti-chicken	Alex 488 (Invitrogen)	1:200
TH	Mouse	MAB318 (Sigma-Aldrich)	1:1000	Horse	anti-mouse	BA2001 (Vector)	1:200
Ox42	Mouse	MCA275G (Serotec)	1:1000	Horse	anti-mouse	BA2001 (Vector)	1:200

### Cell culturing

HeLa cells were cultured in DMEM-Glutamax (Thermo Fisher Scientific 10569010) supplemented with 1% penicillin and streptomycin (Thermo Fisher Scientific 15140122) and 10% FBS (Life Technologies 10270-106). Cells were maintained at 37 °C, 5% CO_2_ and 95% relative humidity. Sub culturing was performed by trypsinizing and then gently washing off cells with fresh media, diluting and seeding in new Nunc EasYFlasks T25 flask (Thermo Fisher Scientific 156367).

### In vitro assays

HeLa cells were seeded in GBO uClear 96 well plates (Greiner Bio-One 781091) at a density of 5000 cells/well in 100 μl culture media. One day after seeding cells, AAVs were diluted in PBS^+/+^ (Gibco 14040-083) to a dilution series ranging from 1 × 10^11^ to 1 × 10^7^ gc/well. The transduced cells were incubated 3 days before fixation with 4% PFA for 20 min. PFA fixed cells were washed once with PBS supplemented with 1 ug/mL DAPI (Sigma-Aldrich D9542) and an additional two washes for 5 min with PBS. Each condition was performed in triplicates.

### Image acquisition

Laser-scanning confocal microscopy was performed on a Leica SP8 confocal microscope at 20 × magnification using 405 nm and 488 nm solid state laser-lines with a standard PMT detector in sequential mode with a pinhole of 1AU. Brightfield images were taken on a Leica DMI8 inverted microscope at 20 × magnification. Overview images from the 96 well plate were taken on a Plate runner HD (Trophos). Striatal overviews for the measurement of autofluorescence intensity were taken with an Olympus VS120 Virtual Slide Scanning Microscope.

### Transmission electron microscopy

Transmission electron microscopy was performed on Athene 400 mesh hexagonal grids, prepared with a Pioloform film and coated with a thin carbon film. Coated grids were glow discharged prior to sample preparation. Virus suspensions were diluted 1:10 in PBS (Gibco 14040-083) and pushed five times through an BD Micro-Fine + 0.3 mL insulin syringe (BD 230-4533) to ensure even suspension of virus particles. Diluted virus suspension was spotted onto prepared grids in 5 µL droplets for 1 min and blotted dry at the edge with Whatman's filter paper. Sample grids were negative stained with 5 µL 2% uranylacetate for 10 s and blotted dry as previously. Analysis of grids were performed on a Technai Biotwin (120KV) electron microscope equipped with a 2 × 2 k veleta Olympus camera. Full and empty capsid particles were counted blinded from randomly selected images^55^.

### Densitometry

For all densitometry measures images were transformed to grayscale-8-bit. Before measurements, ImageJ was calibrated to convert pixel values from grey levels to optical density values. This was done by global calibration of the Rodbard function based on measures from the Kodak No. 3 calibration step tablet. A detailed description of the calibration procedure can be found under: https://imagej.nih.gov/ij/docs/examples/calibration/. The regions of interest were outlined and the grey-pixel intensity value average for the selected region was measured. For the in vitro assay, the values are presented as is as well as a percentage of the DAPI values (GFP/DAPI). For the in vivo assay we performed optical density measures on four regions of the striatum (AP + 1.6, + 0.7, − 0.26, and − 0.92) for GFP-DAB, TH-DAB, and GFP-autofluorescence) and three regions of the midbrain (AP: − 4.5, − 5.2, and − 6.04) for OX42, for the injected (right) and intact (left) side of the brain respectively. For the former only the striatum was outlined whereas for the latter, we compared the left versus the right midbrain.

### TH neuron quantification

We quantified the TH-ir neurons in the SN by counting every single cell from a z-stack merged image encompassing the entire midbrain at the level where the medial terminal nucleus of the accessory nucleus of the optic tract presents a clear visible border between the SN and Ventral Tegmental Area at × 20 magnification, as described previously^62^. TH-ir cell counts on the injected side were expressed as a percentage of the intact side.

### Statistics

For all statistical evaluation, an alpha level of 0.05 was used for determining statistical significance. We used one-way analysis of variances to determine whether the results obtained differed between the experimental groups and a SNK post hoc test to probe significant differences between experimental groups as appropriate. For the comparison between the two groups assessing autofluorescence we used an independent samples *t*-test.

## Results

We excluded four rats from the main analysis due to failed injections (two from the Iod-GFP and two from the Chl-GFP group), assessed by the absence of GFP staining. The remaining group sizes are: Iod/Chl-GFP, both, n = 6; Iod/Chl-noVG, both, n = 8; Iod/Chl-Nil, both, n = 8; PBS, n = 5.

### Both production methods result in similar AAV capsid protein levels and transgene packaging

**Figure 2:**
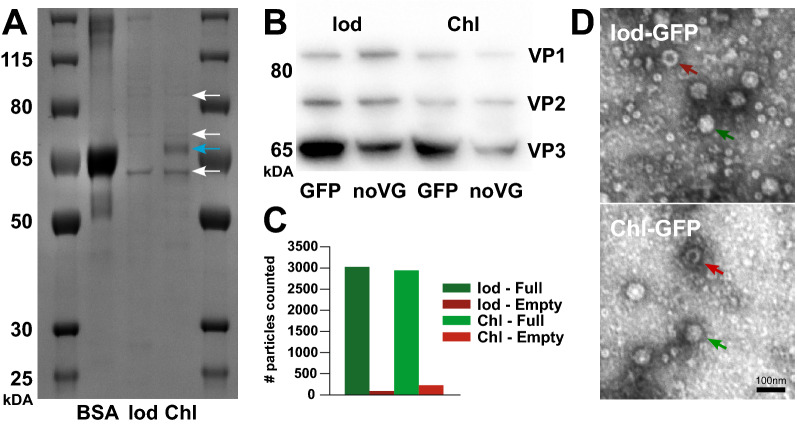
Total protein levels and capsid assessment. (**A**) Coomassie blue gel displaying protein-weight distribution of the two AAV-GFP vectors produced with the Iodixanol-gradient and the Chloroform-precipitation method compared to a BSA control. The bands expected for the three capsid proteins are highlighted with white arrows and the molecular weight for BSA is highlighted with a blue arrow. (**B**) Western-blot for the three capsid proteins VP1, VP2 and VP3 for the four groups containing viral capsids. Figures A and B are cropped to highlight the relevant information. Full sized gels are presented in Supplementary Fig. S1. (**C**) Bar graph displaying the number of full and empty capsid particles counted. (**D**) TEM images displaying empty (red arrows) and full capsids (green arrows) for either production method.

In order to investigate for differences between the two production methods in terms of protein composition, we ran a Coomassie blue staining to assess total protein content and a Western Blot targeting VP1, VP2, and VP3 for capsid composition. When assessing the total protein content in the Coomassie blue staining, we observed similar profiles between the different production methods. We did however observe a pronounced increase in the amount of a band corresponding in size to BSA in the Chl-batch compared to the Iod-batch (Fig. 2A). To confirm that the bands at the respective locations are indeed the three capsid proteins, VP1, VP2, and VP3, we conducted a Western Blot using a specific antibody. The Western Blot (Fig. 2B) showed similar patterns for both production methods, with a stronger band for VP3 compared to VP1 and VP2, consistent with previous studies^63^, indicating a 1:1:10 ratio of the three proteins (data not shown). Quantifications of empty and full AAV capsid particles from transmission electron microscopy (Fig. 2C,D) resulted in 97% and 92% full capsids for the Iod-GFP (full: 3029; empty: 93) and Chl-GFP (full: 2943; empty: 224) groups, respectively.

### Iodixanol- and chloroform-based production methods are equipotent in their ability to transduce a human cell line

**Figure 3:**
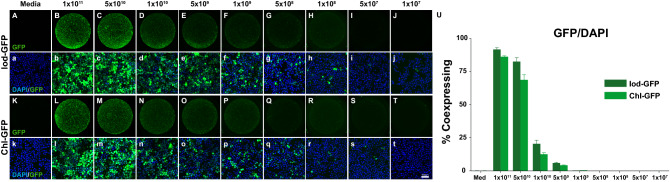
In vitro infectivity assay. Results from the in vitro transduction of HeLa cells for decreasing gc AAV-GFP vector produced from the Iod-GFP (**A**–**j**) and Chl-GFP (**K**–**t**) production methods. Representative low magnification images from the respective groups (Iod-GFP: **A**–**j**, Chl-GFP: **K**–**t**) clearly display the decrease in fluorescence intensity. High magnification confocal microscopy images from the centre of the wells (**a**–**j** and **k**–**t**) indicate that there are a few cells expressing GFP even at relatively low gc-titers. (**U**) Quantifications for actual cell numbers co-expressing DAPI and GFP. Each condition was repeated in triplicates. Note that a conservative threshold was chosen hence underestimating the true transduction rates.

We transduced the human HeLa cell line with a matched, serial dilution of the viral vector stock of both production methods, including the controls. As can be seen in Fig. 3, all wells contained a similar number of cells with 43 975 ± 806 and 46 647 ± 614 cells/well for the Iod and Chl group, respectively (Fig. 3a–t,U). As expected, the number of cells that were GFP positive decreased with a reduction in GFP expressing genome (Fig. 3A–T). Cells in the highest condition using 1 × 10^11^ gc-titer had 37 336 ± 1510 (Iod-GFP) and 38 796 ± 1177 (Chl-GFP) GFP positive cells as quantified by the Metamorph software (Fig. 3U). This corresponds to 91.7% (Iod) and 86% (Chl) of GFP expressing cells, respectively. With half log step dilutions from 5 × 10^10^, 1 × 10^10^, and 5 × 10^9^, the GFP expressing cell numbers decrease from 37,454 ± 1949, 9760 ± 1612, and 2687 ± 348 for the Iod-production and 33,780 ± 1021, 6100 ± 768, and 1987 ± 165 for the Chl-production, respectively. Below a gc-titer of 1 × 10^9^ the expression of GFP was between 0.35% and 0.01%. Cells in neither of the control conditions did express GFP (data not shown).

### Both production methods result in robust transgene expression

**Figure 4:**
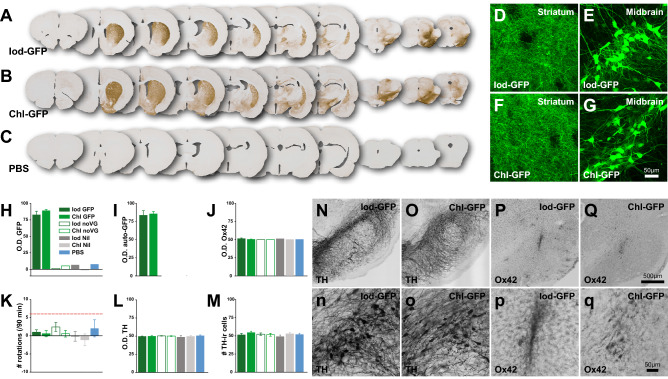
In vivo comparison of production methods on transgene expression and inflammation. Overview of a coronal 1:12 series of the brains labelled for GFP for the two main experimental groups (**A**-Iod-GFP; **B**-Chl-GFP) as well for the PBS-injected control group (**C**). Note that overviews for the four remaining control groups are supplied in the Supplementary Fig. S2). (**D**–**G**) High power confocal microscopy images displaying the strong expression of GFP in the terminal fibres in the striatum (**D**,**F**) and the cell bodies in the substantia nigra (**E**,**G)**. Optical density analysis using ImageJ for the three proteins GFP, Ox42 and TH are presented in (**H**–**J**,**L**), respectively. (**K**) The response to 2.5 mg/kg d-amphetamine was averaged over 90 min. The dotted red line indicates the threshold of well lesioned animals. (**M**) Quantifications of TH-ir cells in the substantia nigra, expressed as percentage of the injected side compared to the uninjected side. Midbrain dopamine cells do not show obvious signs of degeneration (**N**,**o**) and there is only a mild inflammation response at the site of the injection as indicated by the microglial marker Ox42/cd11b (**P**,**q**).

After quantifying GFP expression in vitro we injected the titer-matched vectors from the Iod-GFP and Chl-GFP groups as well as the four control groups into the SNpc of rats which were randomly assigned into one of seven experimental groups. After immunohistochemical staining for GFP in DAB (Fig. 4A–C; Supplementary Fig. S2) we scanned the sections and used ImageJ to perform optical density analysis on four striatal coronal series from each animal. There was strong expression of the GFP transgene only in the Iod-GFP and Chl-GFP groups as expected, with GFP positive fibres in the Striatum (Fig. 4A,B,D,F) and cell bodies in the midbrain (Fig. 4A,B,E,G). There was significant difference in optical density of GFP-DAB stained sections between the seven groups (Fig. 4H; Group, F_6,10_ = 41.418; p < 0.001). Post-hoc analysis revealed that both experimental groups displayed a stronger staining intensity on the injected side versus the control side (Iod-GFP: 82%, Chl-GFP: 89%) compared to the five control groups (< − 1 to 8%; all, p < 0.001). There was no statistical difference in transgene expression between the Iod-GFP and Chl-GFP groups (p = n.s). To exclude the possibility of arbitrarily inflating the signal through the amplification that is caused by the antibody-based immunohistochemistry, we measured the autofluorescence from freshly mounted unstained sections. Similar to the DAB based optical density analysis, we report no difference between the Iod-GFP and Chl-GFP groups (*t*_10_ = 0.298; p = n.s.) in GFP expression (Fig. 4I and Supplementary Fig. S3).

### Neither production method results in preparations that are pro-inflammatory

After assessing the Coomassie blue staining we discovered that the main difference between the two production protocols was in the higher presence of BSA in the Chl-group, which was absent in the Iod-group. To investigate whether the presence of BSA (or any other unknown organic residue in the AAV suspension) could result in neurodegenerative or inflammatory responses, we assessed the integrity of the nigrostriatal dopamine system on a behavioural and immunohistochemical level.

Rats with unilateral degeneration of the nigro-striatal pathway typically respond to a challenge with 2.5 mg/kg d-amphetamine with a strong rotational response that can last for several hours. Although rats can display an inherent directional bias upon dopamine stimulation, well lesioned rats will respond with a strong ipsiversive rotational response of six rotations per minute and higher^58,64^. In the current study we did not find any difference between either group when challenged with amphetamine (Fig. 4K; Group, F_6,42_ = 0.866, p = n.s.). Although some rats did rotate towards one side at low levels, no group reached the levels of rotations that are indicative of a near-complete lesion. Furthermore, staining intensity levels for TH-ir fibres on the intact vs the injected side revealed similar levels of staining intensity (Group average = 49.93% ± 0.41; Group, F_6,42_ = 0.321, p = n.s.; Fig. 4L). When quantifying the number of TH-ir neurons in both the injected and uninjected sides, we did not find differences between the groups (F_6,42_ = 2.203, p = n.s.; Fig. 4M,o). Although there was a small increase in microglial activity at the site of injection, following the tract of the injection needle (Fig. 4P,q), a comparison of staining intensity for the microglial marker Ox42 (cd11b) revealed no significant difference between the injected and the control side for any of the groups (Fig. 4J; F_6,10_ = 4.69, p = n.s.).

## Discussion

A self-complementary AAV2/9 vector encoding for GFP under the CMV promoter was produced according to two different protocols and diluted to 1 × 10^12^ gc/mL. Equal volumes of these titer-matched vectors were injected into the midbrain of naïve adult Sprague–Dawley rats. Six weeks post-AAV transduction the animals were sacrificed, and the brains were processed for GFP-protein expression as well as for neuronal degeneration and microglial activation. From our vector comparison we conclude that our novel AAV production method results in AAV vectors that are equipotent to the gold standard, iodixanol gradient, suggesting that our recently published “small scale” AAV vector production protocol is suitable for widespread in vivo and in vitro applications.

The main difference in the production of the AAV-vectors lies in the harvesting of the vectors. Although our previously published data suggest that our protocol produces AAV-vectors that were functional^7,10–12^, we did not determine whether there are any differences in overall protein content between the two production methods. Different residues during the harvesting steps could possibly result in an inflammatory response and cell loss that is not related to the genetic payload as suggested previously^54^. However, AAV-vectors themselves can cause an immune response, albeit at low levels^65–67^. Hence a thorough investigation is warranted. To assess the overall protein content in the two production methods we conducted a Coomassie Blue gel. The major difference was a higher content of BSA in the chloroform-based protocol. Although BSA is a commonly used additive in cell culture medium, as a major component of FBS, we could not exclude that this protein is causing any adverse effects when injected into the brain. In our evaluation we did not detect any adverse effects on inflammation or neurodegeneration in the timeframe analysed. However, since FBS is not a fully defined component of cell culturing medium, variation between batches may occur. We identified the capsid proteins as bands at their respective weights in the Coomassie Blue gel and confirmed their identity using primary antibodies targeted against the three capsid proteins. As expected, the three capsid proteins VP1, VP2, and VP3 were present in a 1:1:10 ratio, that previously has been described elsewhere^63^.

Although AAVs are rarely used for in vitro studies because of their inability to integrate into the host genome and subsequently being diluted in dividing cell lines, we show that both production methods lead to strong transgene expression without a significant difference in the number of cells expressing GFP, as expected. Although the AAV-vector particles were only effectively and efficiently transducing human cells at high concentrations (> 1 × 10^10^ gc/well), at the highest concentration used (1 × 10^11^ gc/well) nearly every cell displayed GFP expression. Furthermore, whereas the Metamorph cell quantifications returned an overlap of > 85% DAPI/GFP colocalization, the high-power confocal images indicate that the true co-expression rate is higher. This discrepancy is due to the use of a conservative threshold to avoid false positives in the lower gc/well conditions. There are 12 described wild-type capsid variants in the AAV vector toolbox, each of which displays a different tropism and spread^68^. There is also a rapidly increasing number of engineered AAVs, each with unique properties and tropism^10,34,38,69^. In vitro assays based on the correct cell line or cell type can be used as an initial validation for AAV function and gene expression^70^.

After assessing the in vitro capacity of the two AAV vector production methods we focused on assessing whether there were any differences in gene expression levels in vivo, with a focus on transgene expression, cell loss, and neuroinflammatory responses. Although the two methods of producing AAVs differ significantly in the harvesting steps, both vector batches are equipotent in their ability to transduce cells in the midbrain of rats. We could detect strong expression of the transgene in the nigrostriatal pathway and found no difference in GFP expression between the two main experimental groups. One major concern was the possibility of toxicity or the initiation of an immune response due to our novel production method. To that end, we utilized a common behavioural assessment, the amphetamine-induced rotation test. This test has been one of the most reliable tests to assess the unilateral cell loss of the nigro-striatal pathway^58,59^. Although some animals did display a minor rotational side preference, this did not reach the commonly used threshold of six rotations per minute. Only rats that rotate at this rate are commonly considered to display a loss of the nigro-striatal pathway greater than 80%^64,71^. The integrity of the nigro-striatal pathway on the side of injection was furthermore confirmed using a histological analysis. We show that there is no overt cell loss of dopaminergic cells in the midbrain nor a reduction of TH positive terminal projections. Previous studies have shown that overexpression of GFP can cause neurodegeneration by itself, however, only at very high concentrations (3.3 × 10^14^ gc/mL)^72^. Optical density analysis based on the staining intensity did not show any differences between the seven experimental groups. Furthermore, although there was detectable microglial activation at the site of the injection in all groups, this was likely due to the surgical procedure, rather than a difference in the entity used for injection. Overall, based on the data at hand we conclude that both production methods are equipotent on all levels assessed. These results do not, however, exclude that there are differences in the two methods that have not been detected by the current screen. However, whether some organic compounds might be present selectively in either of the two production batches, they did not have any adverse events that emerged in our assessment panel. Another concern is the possibility of a humoral and immune response after systemic injections, or any other route of administration, than the intracerebral injections which were assessed in the present manuscript. It is well established that immune responses can be mounted against the viral capsid or the transgene, both by the innate and adaptive immune system^73,74^. Future comparisons assessing these are imperative.

The main difference between both methods lies in the simplicity of the chloroform-precipitation protocol, which is comparatively faster and does not require specific equipment^51^. The iodixanol based protocol takes longer time and requires specific equipment for purifying and concentrating the virus^53^. Titers resulting from the two production methods are comparable and relates to the choice of capsid rather than the production method itself^10^. The chloroform-based production method has successfully been used to produce a wide variety of AAV serotypes. We, and others, have produced AAV2, AAV2 variants and AAV8^10,75^ as well as AAV5, AAV6 (unpublished data) and AAV9 as presented here. The chloroform-based production of AAV results in reproducible gc-titers between production rounds, fully comparable to those achieved by Iodixanol purification.

The major difference between the two methods is the total yield, where the chloroform-based protocol results in 25–50 μL purified AAV and the iodixanol protocol returns roughly 150–300 μL purified vector. The larger yield also comes with a higher cost. The iodixanol protocol utilizes significantly more flasks, medium, FBS and reagents. As science is driven by economical pressure as well as time pressure, our novel protocol fills that niche on both aspects. For many preclinical studies in the initial phase a variety of capsids, promoters, enhancers, transgenes, and stabilizing domains might be tested before committing to a final construct. Whereas producing 150 μL of a vector at a high gc-titer where only a few microliters are used is not economical, our protocol allows for the fast and economical production of smaller test batches for initial validation. After a final construct is decided upon after evaluations, it makes more economical sense to commit to a large-scale production using the iodixanol-gradient protocol or to order the final vector of interest from a commercial supplier.

In-house production of AAV-vectors can be complicated and usually requires specific equipment^53^. Our recently published protocol for fast and reliable production of high titer AAVs can be implemented in most standardly equipped laboratories^51^. In the current manuscript we have provided data that suggest that our in-house production method to generate high titer AAV-vectors is equipotent in transgene expression and does not lead to neurodegeneration or inflammation in our assessment panel.

## Supplementary Information

Supplementary Information

## Data Availability

All materials and data will be made available on request when possible.
