# Supplementary material for: A comparison of AAV-vector production methods for gene therapy and preclinical assessment
Source: Sci Rep. 2020 Dec 9;10:21532. doi: 10.1038/s41598-020-78521-w (PMC7726153; doi:10.1038/s41598-020-78521-w)
Supplement: Supplementary file 1 — Supplementary Information [file 41598_2020_78521_MOESM1_ESM.pdf]

Supplementary Information

"A comparison of AAV-vector production methods for gene therapy and preclinical assessment "

Marcus Davidsson[1*], Matilde Negrini[2], Swantje Hauser[2], Alexander Svanbergsson[3], Marcus Lockowandt[4], Giuseppe Tomasello[2], Fredric P. Manfredsson[1], Andreas Heuer[2*]

[1]Department of Neurobiology, Barrow Neurological Institute, Phoenix, Arizona, USA
[2]Behavioural Neuroscience Laboratory, Department of Experimental Medical Sciences, Lund University, Lund, Sweden
[3]Neural Plasticity and Repair, Department of Experimental Medical Sciences, Lund University, Lund University, Lund, Sweden
[4]CNS Gene Therapy, Department of Experimental Medical Sciences, Lund University, Lund University, Lund, Sweden
*Corresponding authors: marcus.davidsson@med.lu.se; andreas.heuer@med.lu.se

Supplementary Figure 1

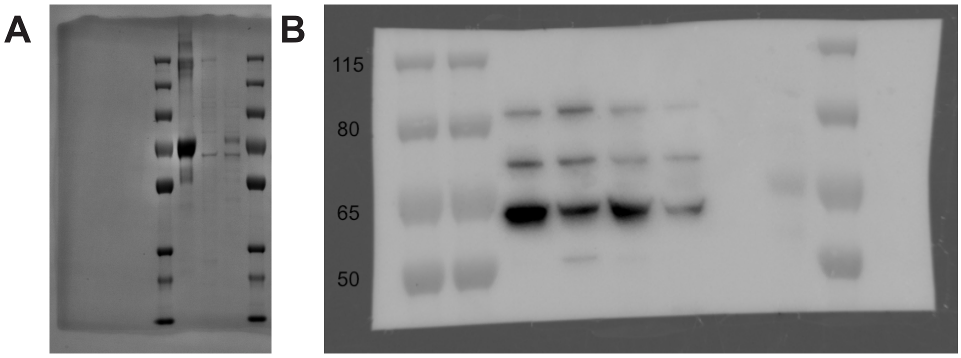

Supplementary Figure 1. *Full length unprocessed images from the Coomassie gel (A) and Western Blot (B) membrane imaged with visible light overlayed ontop of the same membrane imaged with UV to show the MW ladder next to the antibody-bands .*

Supplementary Figure 2

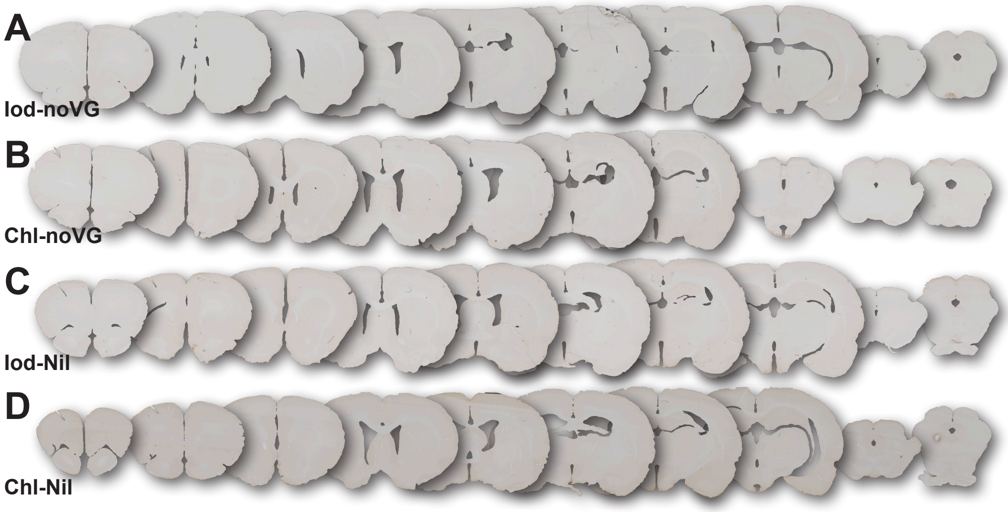

Supplementary Figure 2. *Overview of a coronal 1:12 series of the brains labelled for GFP for the four control groups Iod-noVG (A), Chl-noVG (B), Iod-Nil (C), and Chl-NIL (D).*

Supplementary Figure 3

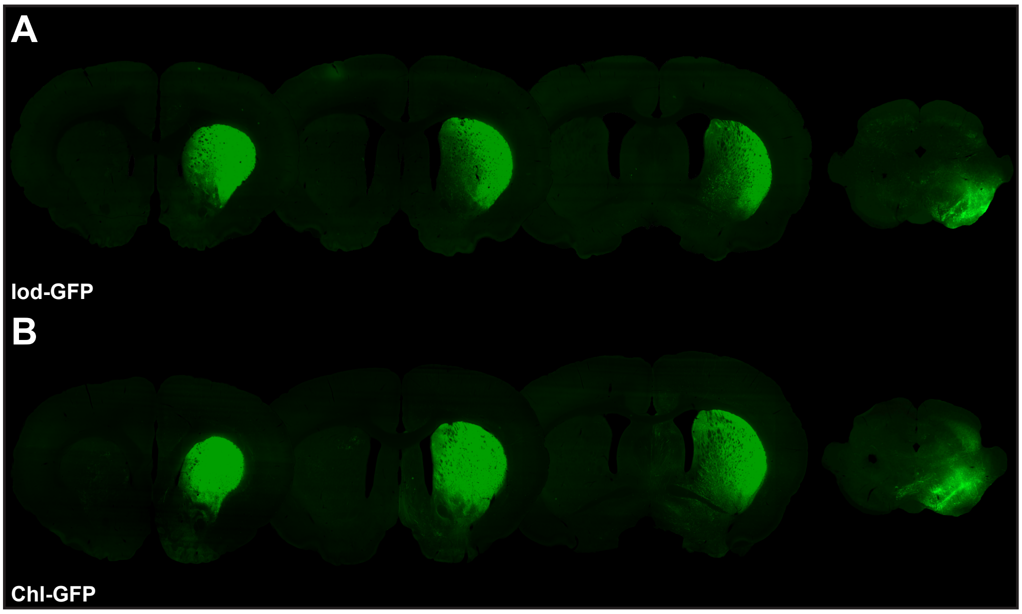

Supplementary Figure 3. *GFP autoflourescence overview of the Iod-GFP (A) and the Chl-GFP (B) experimental groups taken with the Olympus VS120 Virtual Slide Scannining Microscope.*
